# Expression profiling of ETO2-regulated miRNAs in erythroid cells: Possible influence on miRNA abundance^☆^

**DOI:** 10.1016/j.fob.2013.10.004

**Published:** 2013-10-11

**Authors:** Tohru Fujiwara, Yoko Okitsu, Yuna Katsuoka, Noriko Fukuhara, Yasushi Onishi, Kenichi Ishizawa, Hideo Harigae

**Affiliations:** a Hematology and Rheumatology, Tohoku University Graduate School of Medicine, Sendai, Japan; b Molecular Hematology/Oncology, Tohoku University Graduate School of Medicine, Sendai, Japan; c Clinical Research, Innovation and Education Center, Tohoku University Hospital, Sendai, Japan

**Keywords:** ETO2, Erythropoiesis, miRNA, ETO2 (CBFA2T3), core-binding factor, runt domain, alpha subunit 2, translocated to, 3, LMO2, LIM domain only 2, CBF1, core-binding factor 1, RPMI, Roswell Park Memorial Institute, IMDM, Iscove’s Modified Dulbecco’s Media, IL-3, interleukin 3, SCF, stem cell factor, siRNA, small interfering RNA, cDNA, complementary DNA

## Abstract

ETO2 is a component of a protein complex containing master regulators of hematopoiesis, including GATA-1 and SCL/TAL1, and also has RNA binding properties. Although ETO2 has been reported to repress GATA-1 target genes through histone deacetylation of the target gene loci in erythroid cells, little is known about the contribution of ETO2 to microRNA (miRNA) regulation. Here, we conducted miRNA profiling in ETO2-overexpressing and ETO2-silenced K562 cells. The analysis suggests that ETO2 positively regulates the abundance of mature miRNAs, including miR-21, miR-29b and let-7e. Our data suggest a novel mode of ETO2-mediated target gene repression via effects on miRNA expression.

## Highlights

• miRNA profiling was conducted in ETO2-overexpressing and ETO2-silenced K562 cells.
• ETO2 positively regulates the abundance of miRNA.
• ETO2 positively regulates the expression of miR-21, miR-29b and let-7e.

## 1 Introduction

The differentiation of hematopoietic stem cells (HSCs) into specific progenitor cells, and ultimately into diverse blood cell types, is controlled by various transcription factors, represented by the GATA factors [1]. In the context of erythropoiesis, GATA-1 regulates the expression of various erythroid-related genes by recognizing the DNA binding consensus sequence (A/T)GATA(A/G) through dual zinc finger motifs, which are characteristic of the GATA family [2]. GATA-1 forms a complex with another master regulator of hematopoiesis, the basic-helix-loop-helix transcription factor SCL/TAL1 [1,3,4]. SCL/TAL1 is a master regulator of hematopoiesis that binds E-boxes and non-DNA binding components of LMO2, LDB1, and ETO2 [1,3–9].

ETO2 (also known as MTG16 or *CBFA2T3*) confers transcriptional repression mediated by GATA-1–SCL/TAL1 complex through interaction with histone deacetylases (HDACs) [10]. To assess the impact of ETO2 on GATA-1 targets in erythroid cells, we previously conducted mRNA expression profiling analyses and demonstrated that ETO2 represses a relatively small subset of direct GATA-1 targets [11,12]. Beyond the association with GATA–SCL/TAL1 complex, although ETO2 has also been reported to interact with the intracellular domain of Notch1 and the Notch-regulated transcription factor CBF1 [13] as well as T-cell factor 4 (TCF4), which is involved in Wnt signaling [14], the regulatory mechanism mediated by ETO2 remains to be fully elucidated.

The regulation of erythroid differentiation by noncoding RNAs (ncRNAs) is becoming well understood [15]. ncRNAs are broadly classified into housekeeping RNAs (rRNAs, tRNAs, and snoRNAs), small and long ncRNAs (<200 and >200 bases, respectively), siRNAs, PIWI-interacting RNAs (piRNAs), and microRNAs (miRNAs) [15]. Among these ncRNAs, miRNAs are now universally recognized as an extensive and ubiquitous class of regulatory molecules [16]. Several studies demonstrated that numerous miRNAs are induced or repressed during erythroid differentiation [17–21]. GATA-1 directly regulates the expression of miR-144 and miR-451, both of which are intimately involved in the erythroid phenotype [18]. Furthermore, expression data-based correlation analysis revealed that Lmo2–GATA-1 is an important regulatory module for miRNA expression [22]. These findings suggest that GATA-1, or possibly GATA-1–SCL/TAL1 complex, plays an important role in miRNA regulation during erythroid differentiation. Intriguingly, it has also been demonstrated that ETO2 has RNA-binding properties [23], suggesting that ETO2 may regulate its targets by affecting an RNA component in erythroid cells.

The present study was performed to improve our knowledge regarding the role of ETO2 in miRNA regulation in erythroid cells using the K562 erythroleukemia cell line and cord blood CD34-positive cell-derived primary erythroblasts.

## 2 Materials and methods

### 2.1 Cell culture

Human K562 erythroleukemia cells were maintained in RPMI-1640 medium containing 10% fetal bovine serum (Biowest) and 1% penicillin–streptomycin (Sigma). To obtain primary erythroblasts, cord blood CD34-positive cells were suspended into erythroid IMDM medium containing IL-3 (50 ng/mL), SCF (50 ng/mL), and erythropoietin (EPO) (2 IU/mL) [12]. The use of cord blood samples for the study was approved by the ethics committee of Tohoku University. Ethical considerations according to the declaration of Helsinki were followed.

### 2.2 Gene transfer and vectors

ETO2 mRNA was cloned into pcDNA(−)/Myc-His vector (Invitrogen). ETO2 overexpression in K562 cells was conducted with Amaxa Nucleofector kit solution V (Amaxa Biosystems), as described previously [12]. For ETO2 overexpression in cord blood CD34-positive cell-derived primary erythroblasts, retroviral vector (pBABE-puro) was used as described previously [12]. Briefly, CD34-positive cells were incubated with the viral supernatant for infection, and the cells were differentiated for 4 days with erythroid medium containing 1 μg/mL puromycin (Sigma) for selection of transduced cells.

### 2.3 Silencing of gene expression by siRNA

For siRNA-mediated transient human ETO2 knockdown in K562 cells, siGENOME human *CBFA2T3* siRNA – SMART pool (Thermo Scientific Dharmacon) was used. The antisense sequences of the siRNA for human *CBFA2T3* were UGAAUGAGGUGAAGCGGCA, CAGCGGAGGUGAAGACGCA, CCAAAGAGAACGGGUCAGA, CAUUGACGAUCGAGGAGUU. As a negative control, siGENOME non-targeting siRNA pool #1 (Thermo Scientific Dharmacon) was used. siRNA was transfected into K562 cells using Amaxa Nucleofector kit solution V with the T-016 program (Amaxa Biosystems). siRNA was transfected twice at 0 and 24 h, and the cells were harvested at 48 h.

### 2.4 Real-time quantitative RT-PCR

Total RNA was used to synthesize cDNA with Superscript II (Invitrogen) as described previously [7,10,12]. Product amplification was monitored by measuring SYBR Green fluorescence and normalized relative to *GAPDH* mRNA. Primer sequences were as follows; *ETO2* Forward: 5′-ATTGACGATCGAGGAGTTTCAT-3′, Reverse: 5′-GCAGCAAGGGCAGGTTT-3′, *GAPDH* Forward: 5′-GAAGGTCGGAGTCAACGGATTT-3′, Reverse: 5′-GAATTTGCCATGGGTGGAAT-3′.

For quantification of miRNA expression, cDNA was synthesized from total RNA using a miRCURY LNA™ Universal cDNA synthesis kit (EXIQON), and subsequently analyzed with miRCURY LNA™ PCR primer set and SYBR Green master mix according to the manufacturer's instructions (EXIQON). The primers used in the study were; hsa-miR-21 (MIMAT0000076), hsa-miR-29b (MIMAT0000100), hsa-let-7e (MIMAT0000066), hsa-miR-19b-1* (MIMAT0004491), hsa-miR-20a (MIMAT0000075) and hsa-miR-15b (MIMAT0000417). Product accumulation was monitored by measuring SYBR Green fluorescence and normalized relative to the RNA spike-in (UniSp6 CP), and changes in expression were quantified by the ΔΔ threshold cycle (ΔΔCγ) method. Control reactions lacking cDNA synthase yielded little to no signal.

### 2.5 miRNA profiling analysis and miRNA target prediction analysis

Human miRNA Oligo chip V16.1.0.0 (Toray) was used according to the manufacturer's protocol. For global normalization, background value was subtracted, and subsequently adjusted to the average signal value of 25.

### 2.6 Western blotting analysis

Western blotting analysis was conducted as described previously [7,12].

### 2.7 Antibodies

Antibodies to ETO2 (C-20) and α-Tubulin (CP06) were obtained from Santa Cruz Biotechnology and Calbiochem, respectively.

### 2.8 Statistics

Statistical significance was assessed by two-sided Student's *t* test. In all analyses, *P*  < 0.05 was taken to indicate statistical significance.

## 3 Results and discussion

**Fig. 1:**
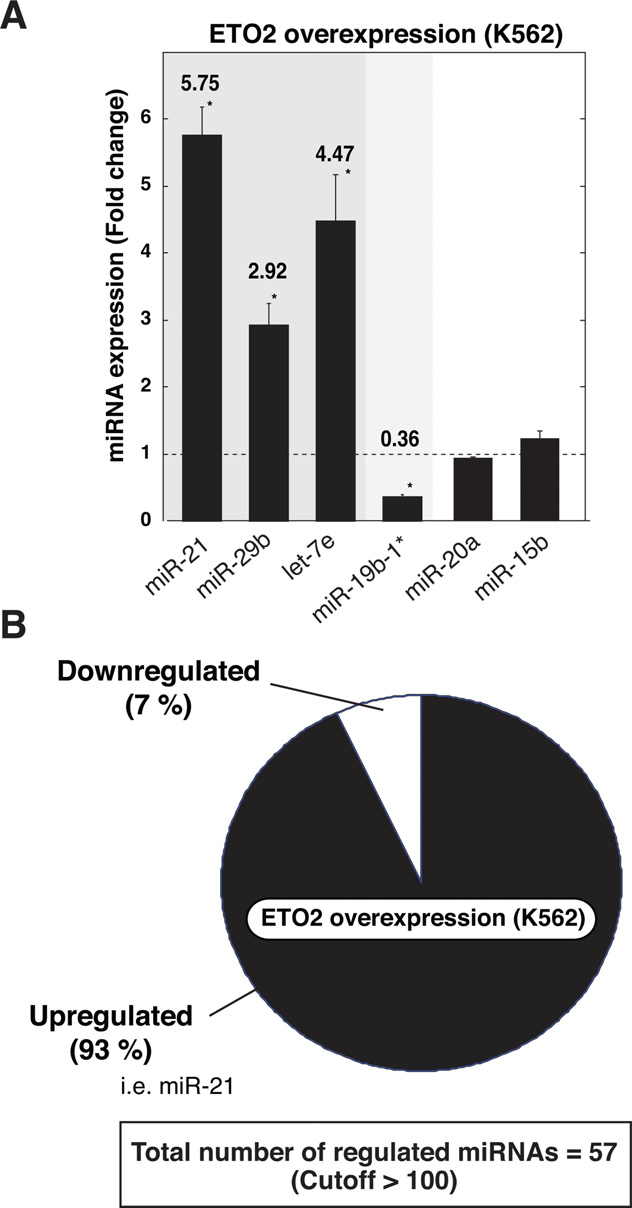
miRNA profiling in ETO2-overexpressing K562 cells. (A) Quantitative RT-PCR validation of array results for ETO2-regulated miRNAs (Suppl. Table 1). The data are expressed as means ± SE (*n* = 6). *P < 0.05. (B) Pie chart analysis denoting the percentage of upregulated or downregulated miRNAs after ETO2 overexpression. We defined >1.5-fold changes and an expression cutoff >100. Upregulated miRNA included miR-21.

We conducted miRNA profiling using identical RNA samples from ETO2-overexpressing K562 cells as described previously [12]. The analysis revealed that ETO2 overexpression upregulated and repressed 182 and 51 miRNAs (>1.5-fold), respectively (Suppl. Table 1). Unexpectedly, we did not observe significant expression changes in miRNAs implicated in erythroid differentiation, such as miR-150 (which affects Myb) [24], miR-223 (LMO2) [25], miR-191 (Riok3 and Mxi1) [26], as well as GATA-1-regulated miR-144 and 451 [18] (Suppl. Table 1). Quantitative RT-PCR-based validation analysis demonstrated significant upregulation of miR-21, miR-29b and let-7e, and downregulation of miR-19b-1*, whereas miR-20 and miR-15b were unaffected by ETO2 overexpression (Fig. 1A). Interestingly, if we set the cutoff value >100 based on miRNA expression level, ETO2 overexpression downregulated the expression of only 7.0% of mature miRNAs (Fig. 1B).

**Fig. 2:**
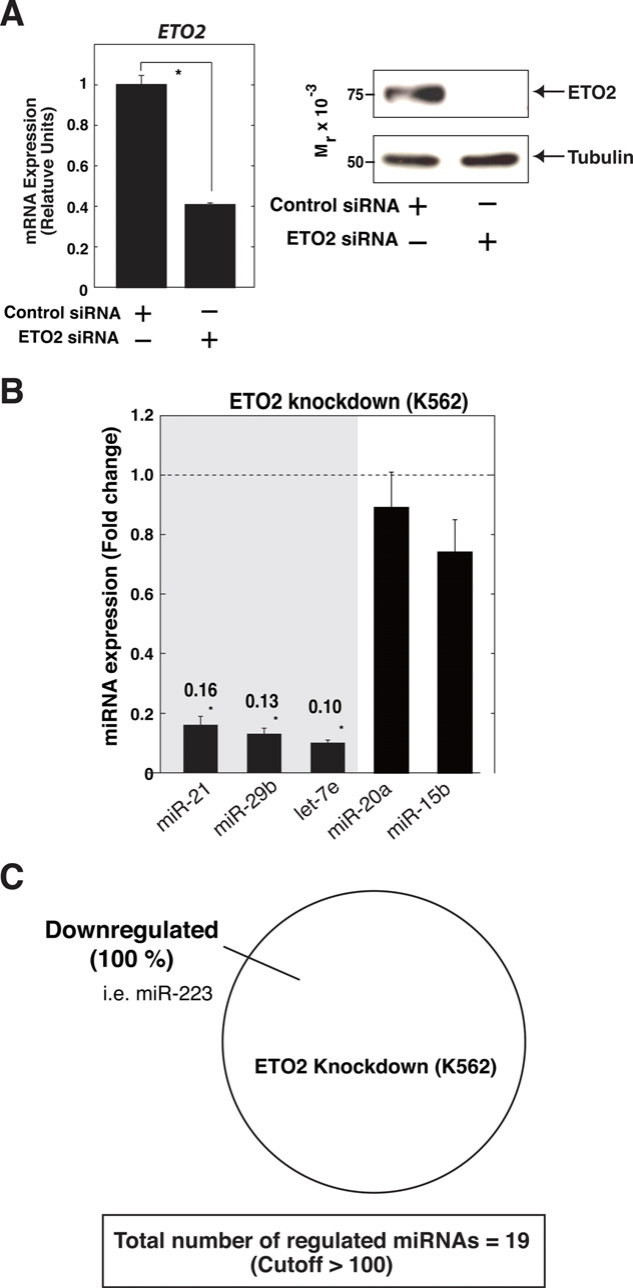
miRNA profiling in ETO2-silenced K562 cells. (A) Quantitative RT-PCR (left) and Western blotting of whole-cell extracts (right) in ETO2 knockdown K562 cells. Anti-ETO2 antibody was used for the analysis. The expression of *ETO2* relative to that of *GAPDH* was calculated (*n* = 3, mean ± SE). α-Tubulin was used as a loading control. *P < 0.05. (B) Quantitative RT-PCR analysis for ETO2-regulated miRNAs (Fig. 1A). The data are expressed as means ± SE (*n* = 6). *P < 0.05. (C) Pie chart analysis indicating the percentage of upregulated or downregulated miRNAs after ETO2 knockdown. We defined >1.4-fold changes and an expression cutoff >100. Downregulated miRNA included miR-223.

We next conducted siRNA-mediated ETO2 knockdown in K562 cells (Fig. 2A), and confirmed significant downregulation of miR-21, miR-29b, and let-7e by ETO2 knockdown (Fig. 2B), suggesting that ETO2 positively regulates the expression of these miRNAs. In addition, miRNA profiling analysis identified miR-223 among the downregulated miRNA ensemble (1.7-fold, Suppl. Table 2), which was reported to inhibit LMO2 expression by directly binding to LMO2 3′-untranslated region (3′-UTR) [25]. As ETO2 level decreases with erythroid differentiation [12], the decrease may contribute to the reduction of miR-223 level, leading to increased LMO2 expression to promote erythroid differentiation [25]. The results of miRNA profiling analysis indicated that ETO2 knockdown upregulated and repressed 2 and 105 miRNAs (>1.4-fold), respectively (Suppl. Table 2). Furthermore, if we set the cutoff value >100 based on miRNA expression level, ETO2 knockdown exclusively downregulated miRNAs (Fig. 2C), which supports the results of ETO2 overexpression (Fig. 1). Taken together, our results suggest that ETO2 positively regulates the abundance of mature miRNAs.

As ETO2 is known as a transcriptional corepressor [7–12], it is difficult to consider that the ETO2-associated miRNA abundance was attributable to the consequences of ETO2-mediated direct transcriptional activation of target pre-miRNA transcripts. On the other hand, a previous study indicated that overexpression of HDAC1 downregulated only 9.0% of regulated miRNAs [27], which was similar to our observations with ETO2 overexpression (Fig. 1). This previous study also indicated that HDAC1 enhances miRNA processing via deacetylation of Drosha/DGCR8 [27]. Thus, based on the observations that ETO2 directly associates with HDAC1 [10] and exerts RNA binding properties [23], we speculate that ETO2 may promote processing of a subset of miRNAs, leading to transcriptional/translational repression. However, we cannot exclude the possibility that ETO2 directly represses other factors, which might lead to the increased pre-miRNA transcripts, which warrants further analyses. Taken together, our data suggest a novel mode of ETO2-mediated target gene repression by affecting miRNA.

**Fig. 3:**
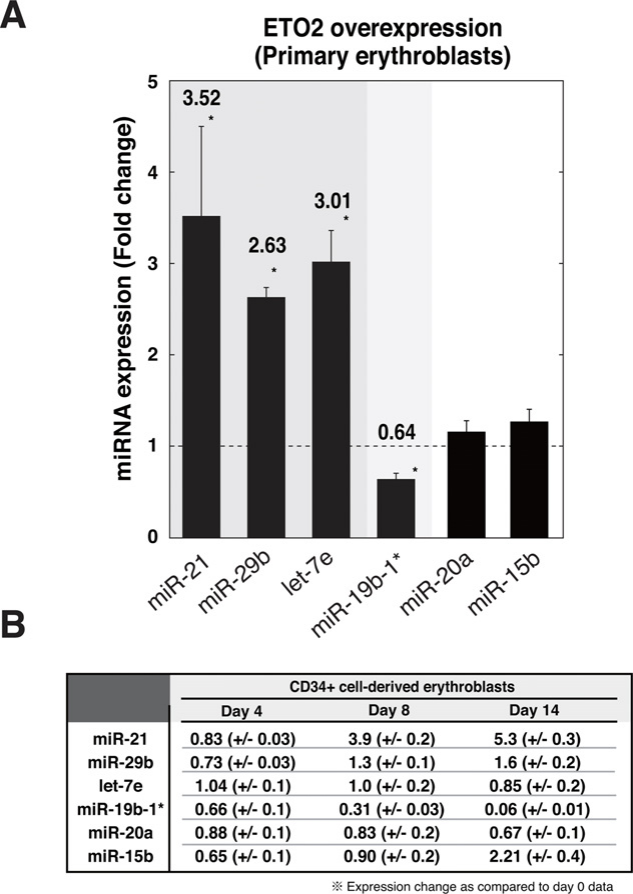
Quantitative RT-PCR analysis for ETO2-regulated miRNAs in primary erythroblasts. (A) Quantitative RT-PCR validation of array results for ETO2-regulated miRNAs (Figs. 1A and 2B), using the identical RNA sample sets of primary erythroblasts overexpressed with ETO2 [12]. The data are expressed as means ± SE (*n* = 6). *P < 0.05. (B) The table summarizes changes in expression of genes on days 4, 8 and 14 in CD34+ cell-derived primary erythroblasts (mean ± SE, *n* = 3), using the identical RNA sample sets of primary erythroblasts [12]. The changes in miRNA expression were quantified by the ΔΔ threshold cycle (ΔΔCγ) method, and presented as fold changes as compared to day 0.

We demonstrated that ETO2 positively regulates the expression of miR-21, miR-29b, and let-7e based on the K562 erythroid cell line (Figs. 1 and 2). However, the phenotypic and karyotypic differences between K562 cells and normal erythroblasts limit the importance of our findings. Therefore, we also confirmed these findings based on human cord blood CD34-positive cell-derived primary erythroblasts. We used the identical RNA sample sets as used in our previous study, which demonstrated that ETO2 overexpression in primary erythroblasts significantly decreases representative erythroid-related genes, such as *HBB*, *HBA*, and *ALAS2* [12]. As shown in Fig. 3A, ETO2 overexpression resulted in significant upregulation of miR-21, miR-29b, and let-7e, and downregulation of miR-19b-1*, whereas miR-20 and miR-15b were constant, which supports the possibility that it may be a universal or physiological phenomenon that ETO2 regulates these miRNAs in erythroid cells. Furthermore, we next examined the expression of these miRNA during erythroid differentiation from CD34-positive cells, based on the identical RNA sample [12]. As shown in Fig. 3B, we observed the modest upregulation of miR-21 and miR-15b, while the obvious downregulation of miR19b-1*, during erythroid differentiation. As ETO2 protein level peaked at day 4 and almost diminished at the later stage of differentiation [12], the decrease of miR-19b-1* at day 4 was consistent with our observations (Fig. 3B). However, the reason why the expressions of miR-21, miR-29b and let-7e at day 4 were constant remains unknown (Fig. 3B). We speculate that ETO2-independent regulatory mechanism might affect the expression of these miRNAs during erythroid differentiation. Nevertheless, ETO2-mediated upregulation of miR-21, miR-29b, and let-7e may have some important roles during erythroid differentiation.

miR-21 and miR-29b have been reported to be involved in the development of lymphoma/leukemia as well as cancer drug resistance [28–30], whereas their roles in erythropoiesis have remained elusive. Recent evidence suggested that increased levels of miR-21 are seen in patients with myelodysplastic syndrome (MDS), and treatment with miR-21 inhibitor promotes erythropoiesis based on the mouse model of bone marrow failure syndrome as well as *in vitro* analysis with primary MDS bone marrow progenitors [31]. The finding suggests that ETO2 inhibits erythroid differentiation partly by increasing miR-21 level. In the context of the role of ETO2 in hematological malignancies, ETO2 may be involved in the pathogenesis of MDS. In addition, AML1–ETO2 fusion protein resulting from t(16;21)(q24;q22) translocation has been associated with myeloid malignancies [32]. As the fusion protein consists of the N-terminus region of AML1 and almost the entire coding region of ETO2 [32], the chimeric protein may affect miR-21 and miR-29b levels. On the other hand, the upregulation of miRNA cluster 99b/let-7e/125a suggests a possible role of ETO2 in hematopoietic stem cell function and erythroid differentiation [33,34].

In summary, our data suggest that ETO2 regulates the abundance of mature miRNAs in erythroid cells. In addition to conferring histone deacetylation of the target gene loci, ETO2 may contribute to the repression of target genes by affecting miRNA levels. We consider that these regulatory mechanisms may target networks of genes and signaling pathways to accomplish the erythroid differentiation program.

## Disclosures

The authors have no conflicts of interest to disclose.

## Appendix Supplementary materials

Supplementary Table 1. miRNA profiling of ETO2-overexpressing K562 cells.

Supplementary Table 2. miRNA profiling of ETO2-silenced K562 cells.
